# Supplementary material for: Photosynthetic light requirement near the theoretical minimum detected in Arctic microalgae
Source: Nat Commun. 2024 Sep 4;15:7385. doi: 10.1038/s41467-024-51636-8 (PMC11375000; doi:10.1038/s41467-024-51636-8)
Supplement: Supplementary file 1 — Supplementary Information [file 41467_2024_51636_MOESM1_ESM.pdf]

Supplementary Information

Photosynthetic light requirement near the theoretical minimum detected in Arctic microalgae

Clara J.M. Hoppe*[1], Niels Fuchs[2], Dirk Notz[2], Philip Anderson[3], Philipp Assmy[4], Jørgen Berge[5], Gunnar Bratbak[6], Gaël Guillou[7], Alexandra Kraberg[1], Aud Larsen[8], Benoit Lebreton[7], Eva Leu[9], Magnus Lucassen[1], Oliver Müller[7], Laurent Oziel[1], Björn Rost[1,10], Bernhard Schartmüller[6], Anders Torstensson[11,12], Jonas Wloka[13]

[1]Alfred-Wegener-Institut - Helmholtz-Zentrum für Polar- und Meeresforschung, Bremerhaven, Germany

[2]Center for Earth System Sustainability, Institute of Oceanography, Universität Hamburg, Germany

[3]Scottish Association for Marine Science, Oban, Scotland

[4]Norwegian Polar Institute, Fram Centre, Tromsø, Norway

[5]UiT The Arctic University of Norway, Tromsø, Norway

[6]University of Bergen, Bergen, Norway

[7]Joint Research Unit Littoral, Environment and Societies (CNRS - University of La Rochelle), La Rochelle, France

[8]NORCE Norwegian Research Centre, Bergen, Norway

[9]Akvaplan-niva, Fram Centre, Tromsø, Norway

[10]Faculty of Biology/Chemistry, University Bremen, Bremen, Germany

[11]Department of Aquatic Sciences and Assessment, Swedish University of Agricultural Sciences, Uppsala, Sweden

[12]Swedish Meteorological and Hydrological Institute, Community Planning Services - Oceanography, Västra Frölunda, Sweden

[13]Independent Researcher, Bremen, Germany

*Correspondence: Clara.Hoppe@awi.de

Supplementary Note 1

Change Point Analysis

In order to statistically estimate the change point at which Chlorophyll *a* (Chl-a) started to accumulate, we used the R package "Detection of Structural Changes in Climate and Environment Time Series" (EnvCPT[1,2]; v1.1.3). We included 8 statistical models in our analysis: a constant mean, a constant linear trend, a constant mean with first-order autocorrelation, a constant linear trend with first-order autocorrelation, multiple change points in the mean, multiple change points in the linear trend, multiple change points in the mean with first-order autocorrelation, and multiple change points in the linear trend with first-order autocorrelation (Figure S11). All model fits are ranked with the Akaike Information Criterion (AIC, Figure S11), which combines the maximum likelihood estimate for each statistical model, i.e., the optimal parameter set to describe the time series. The statistical model with the lowest AIC score corresponds to the model that is best suited to describe changes in the given time series. Applying this methodology to the chlorophyll *a* time series, with a minimum segment length of 5 days in the analysis to minimize the impact of day-to-day variability, we found that 6 of the 8 models returned successful fits, while the other two failed due to the small size of the dataset. The "multiple change in the mean"-model was the most effective model for describing the time series, although all models considering changes in the mean and/or trend provided satisfying results with all models identifying the first change point between the 26th and 28th of March. A secondary changepoint was consistently detected at the end of April. This demonstrates that the result is robust and not sensitive to the choice of the statistical model, as multiple changes in the time series were consistently detected.

Supplementary Figures and Tables

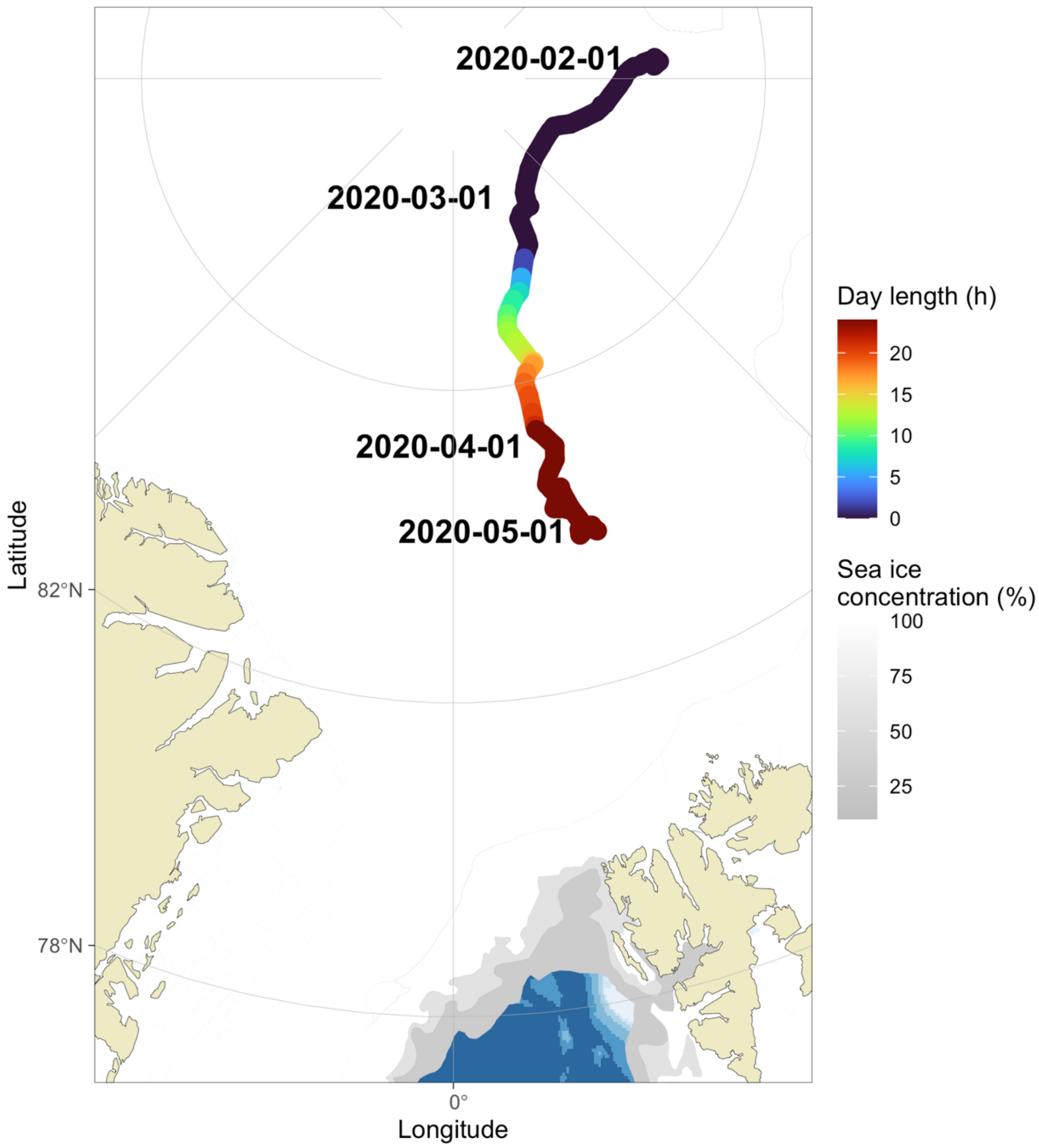

Figure S1: Map of the drift in the Central Arctic Ocean.

Map indicating the drift track of RV *Polarstern* during the study period from February to May 2020 as part of the year-long MOSAiC drift[3]. Coloration of the drift track indicates day length (sun over the horizon) in hours. Background gray scales indicate maximum sea ice concentrations in 2020 (on 2020-03-06).

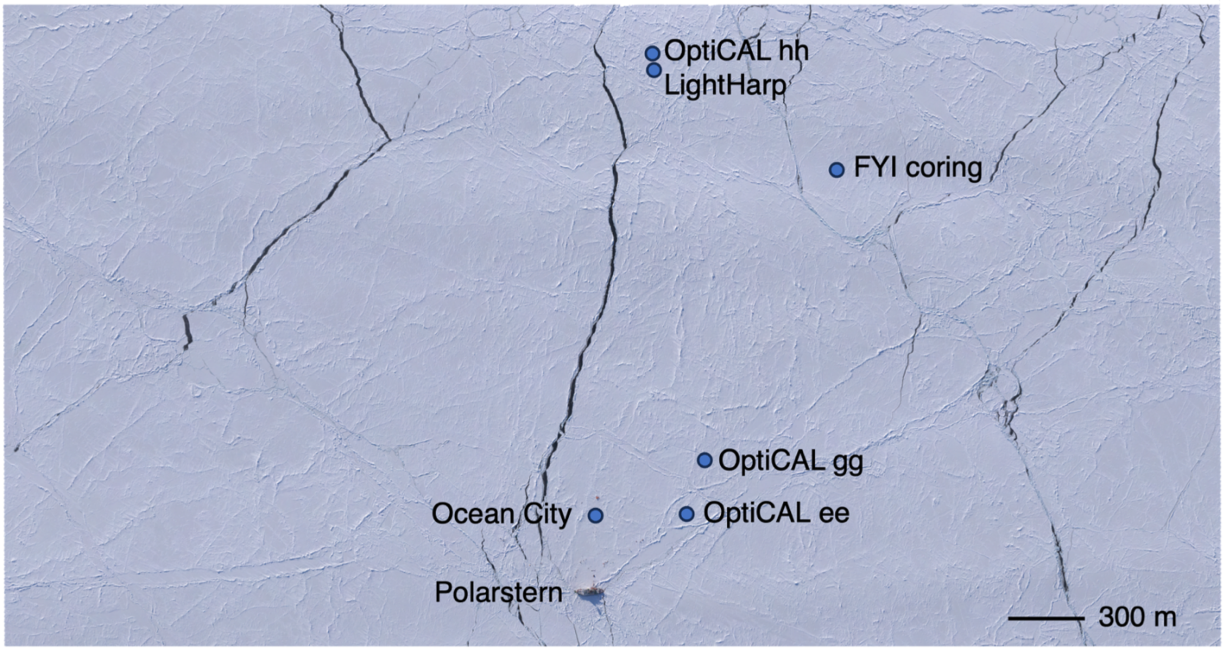

Figure S2: Aerial photograph of the MOSAiC study area including all sampling sites. Orthomosaic from georeferenced aerial photographs of the MOSAiC study area taken with a helicopter on April 23rd 2020 (for methods see Fuchs[4]). Sampling sites relevant for this study are water sampling at 20 m depth via CTD rosettes deployed through holes in the ice at Ocean City and next to RV *Polarstern* (see Rabe et al.[5]), and water sampling from the ship's underway system at 11 m depth. Sea ice cores for sea ice Chl-a and Net Primary Production (NPP) were collected at the first year ice (FYI) coring site. Light measurements were collected with OptiCALs gg, hh, ee down to 50m50 m depth as well as the light harp inside the ice column only. Approximate distances are indicated by the 300 m scale bar. Stitched orthomosaic produced by one of the co-authors from aerial photographs collected during the MOSAiC expedition by the Alfred Wegener Institute.

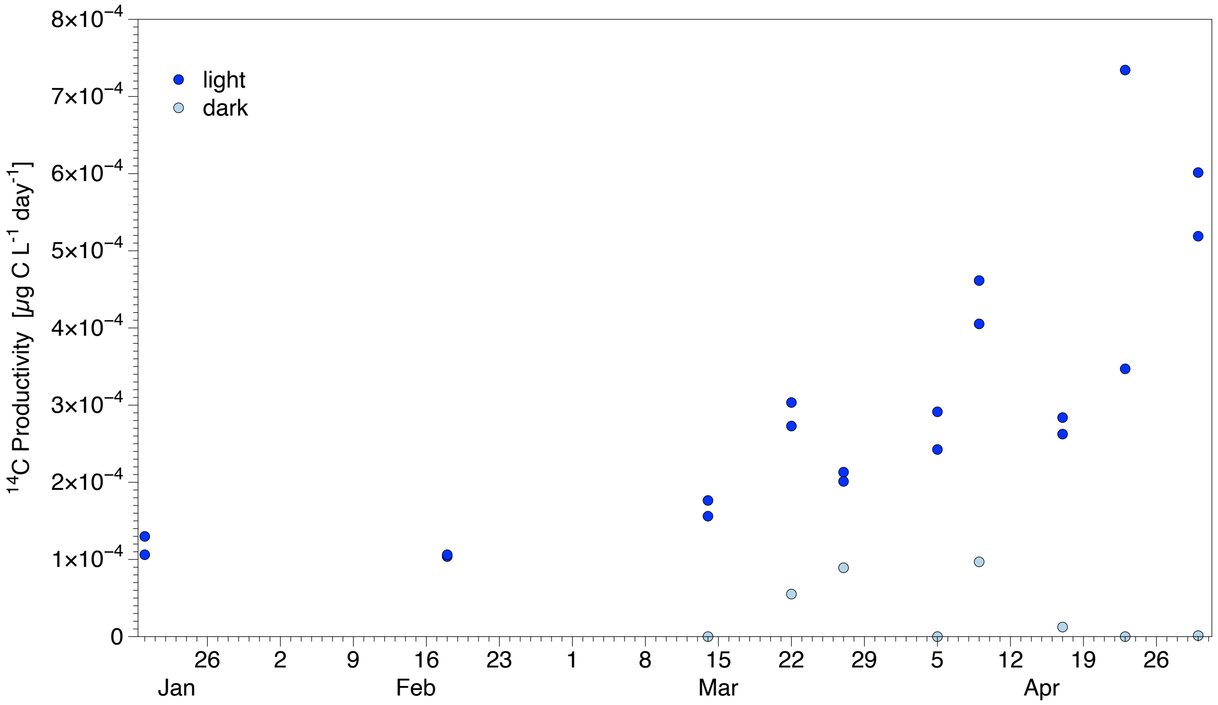

Figure S3: ^{14}C primary productivity from light and dark incubations.

Potential ^{14}C primary productivity as a proxy for potential net primary production rates from dark (light blue) and light incubations (dark blue) under reference conditions in the laboratory (1°C, 10 µmol photons m^{-2} s^{-1}) measured in water samples from 20 m water depth sampled via CTD-rosette casts as a function of time.

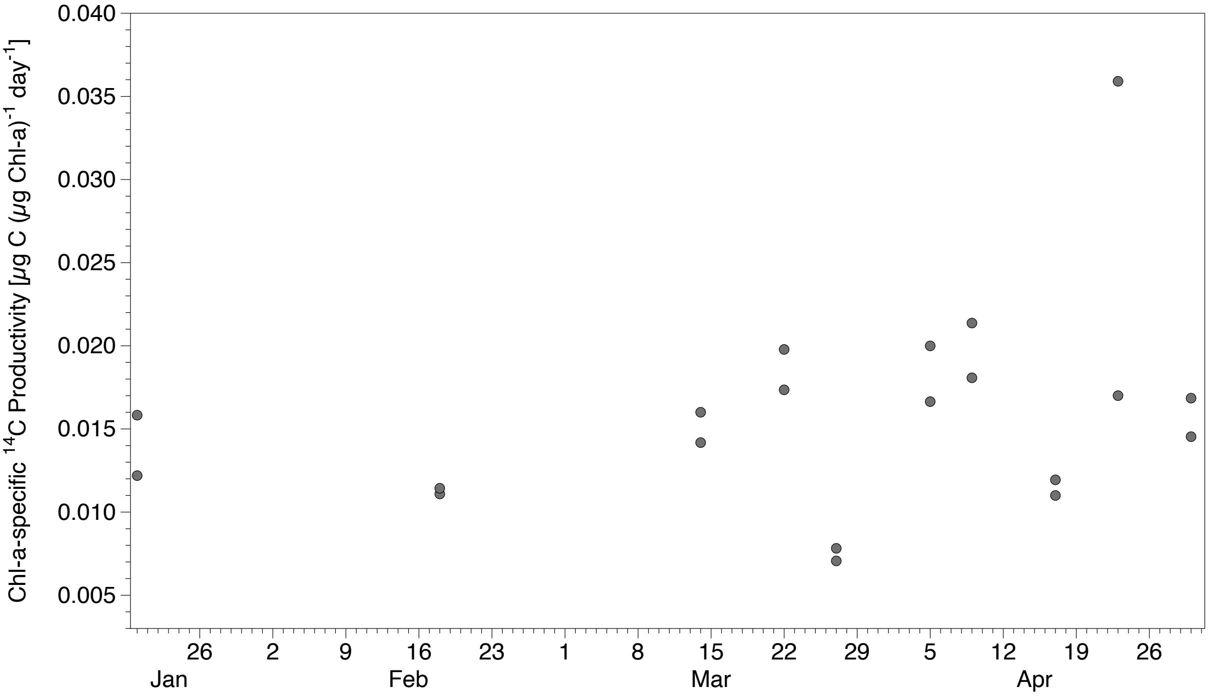

Figure S4: Chl-a-specific carbon fixation rates from the water column.

Chl-a-specific potential ^{14}C productivity as a proxy for potential net primary production rates under reference conditions in samples from 20 m water depth as a function of time. The lack of a trend in the data (Mann-Kendall Trend test: S=15, p value = 0.11) indicates that changes in NPP are driven by biomass changes and not by photophysiological processes.

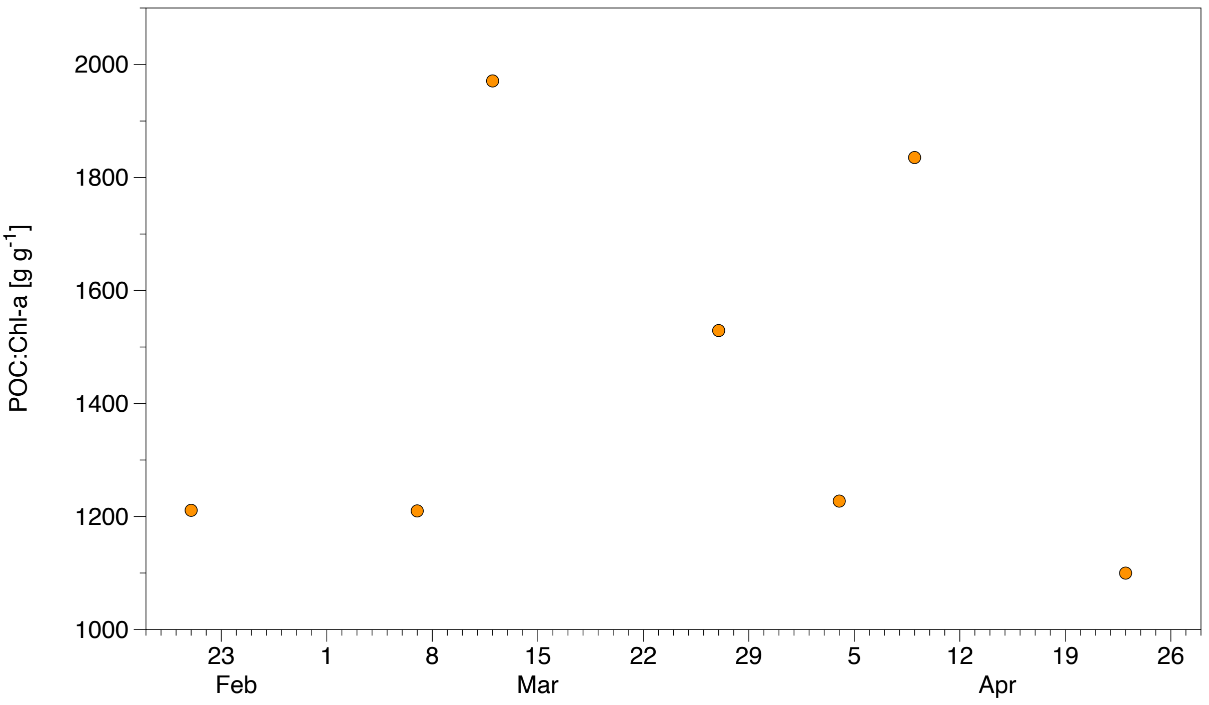

Figure S5: Temporal evolution of the ratio between particulate organic carbon and Chl-a from surface waters.

Ratios of particulate organic carbon (POC) to Chl-a (POC:Chl-a) measured in samples from 20 m water depth as a function of time. The lack of a trend in the data (Mann-Kendall Trend test: S=-1, p-value = 0.5) indicates that changes in Chl-a concentrations are driven by phototrophic biomass changes and not by photophysiological processes.

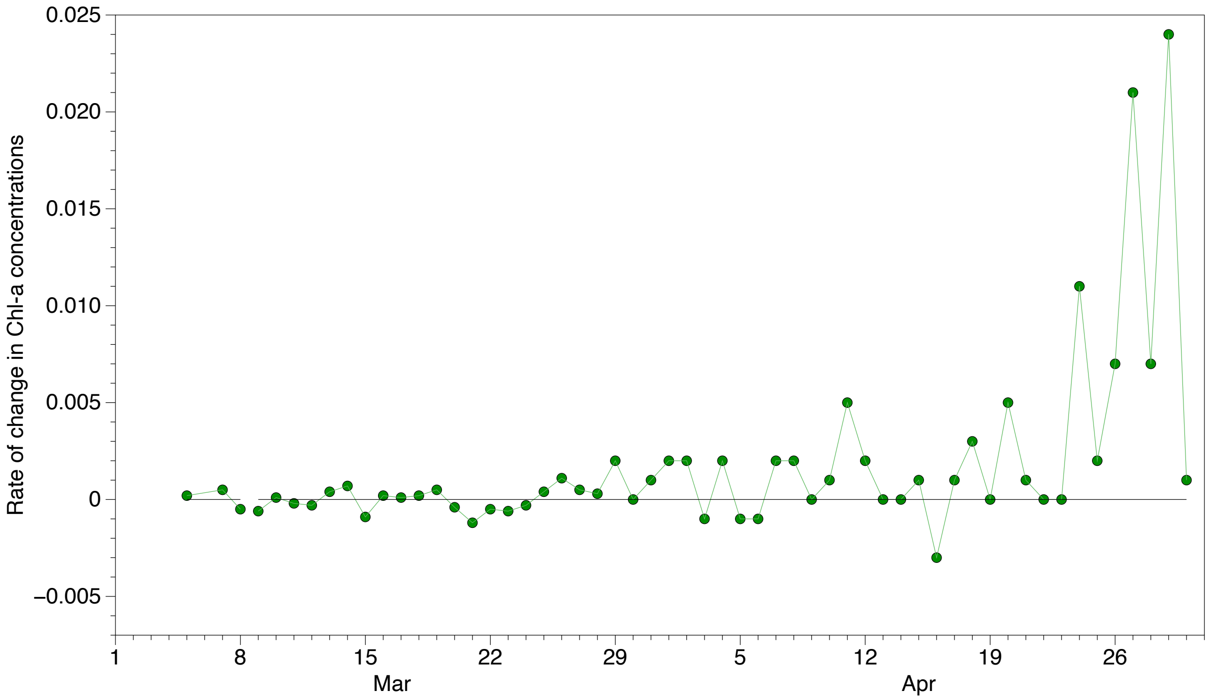

Figure S6: Rate of change in surface Chlorophyll a concentrations.

Rate of change in surface Chl-a concentrations from 11 m water depth over a 5-day period as a function of time. Several consecutive positive values indicate a consistent increase in Chl-a derived biomass.

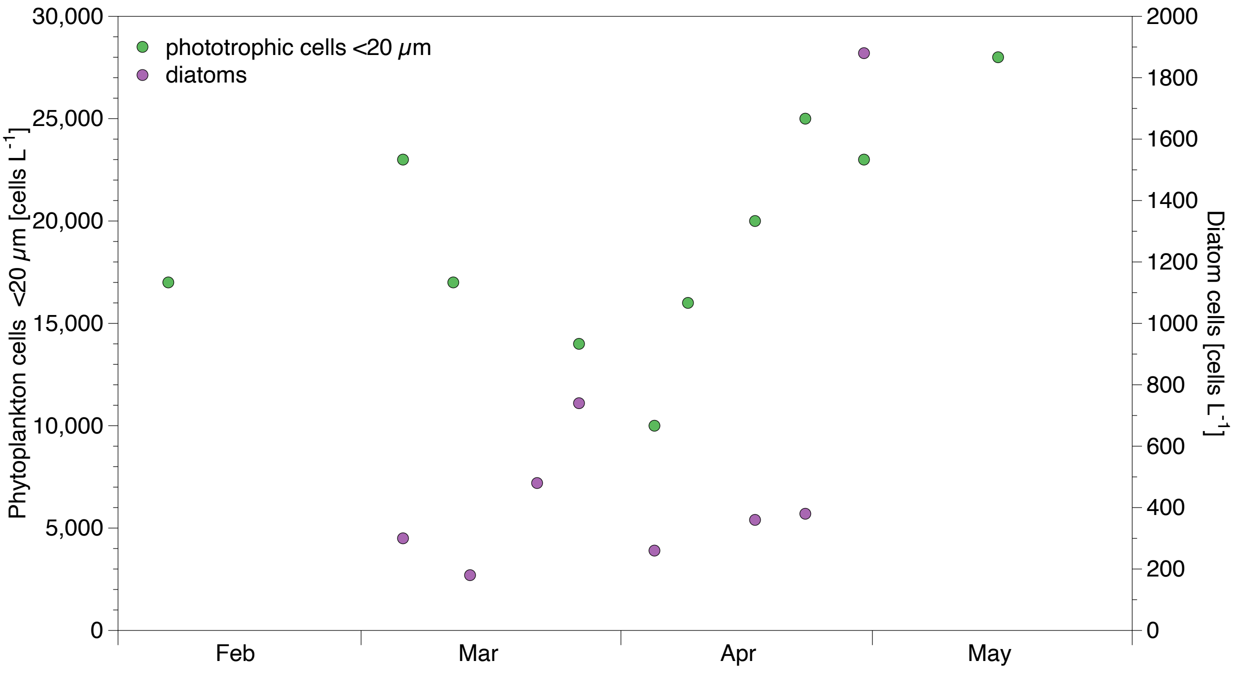

Figure S7: Temporal evolution of water column cell counts.

Flow cytometric counts of phytoplankton cells in the size-range of 1 to 20 µm (green symbols) and of diatom cells identified by light microscopy (purple symbols) from 20 m water depth as a function of time.

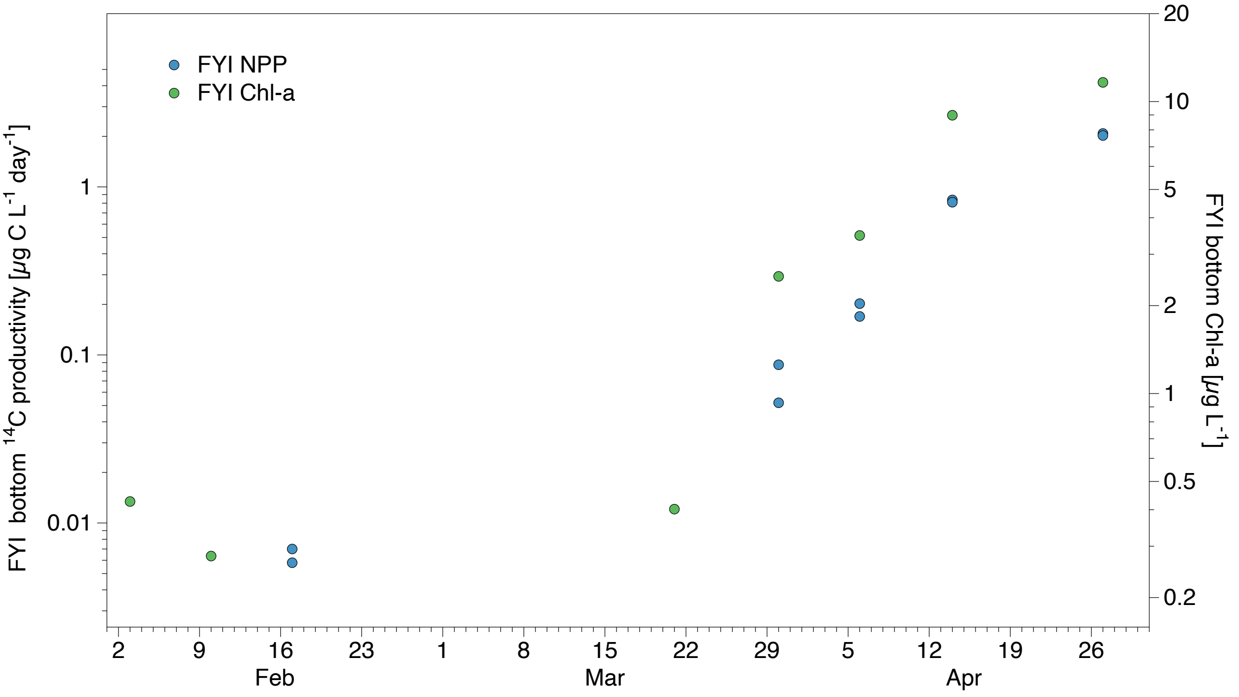

Figure S8: Temporal evolution of bottom ice biomass build-up and primary production. Potential ^{14}C productivity as a proxy for net primary production (NPP; blue symbols), and Chl-a concentrations (green symbols) of bottom 5 cm sections from first year sea ice (FYI) as a function of time.

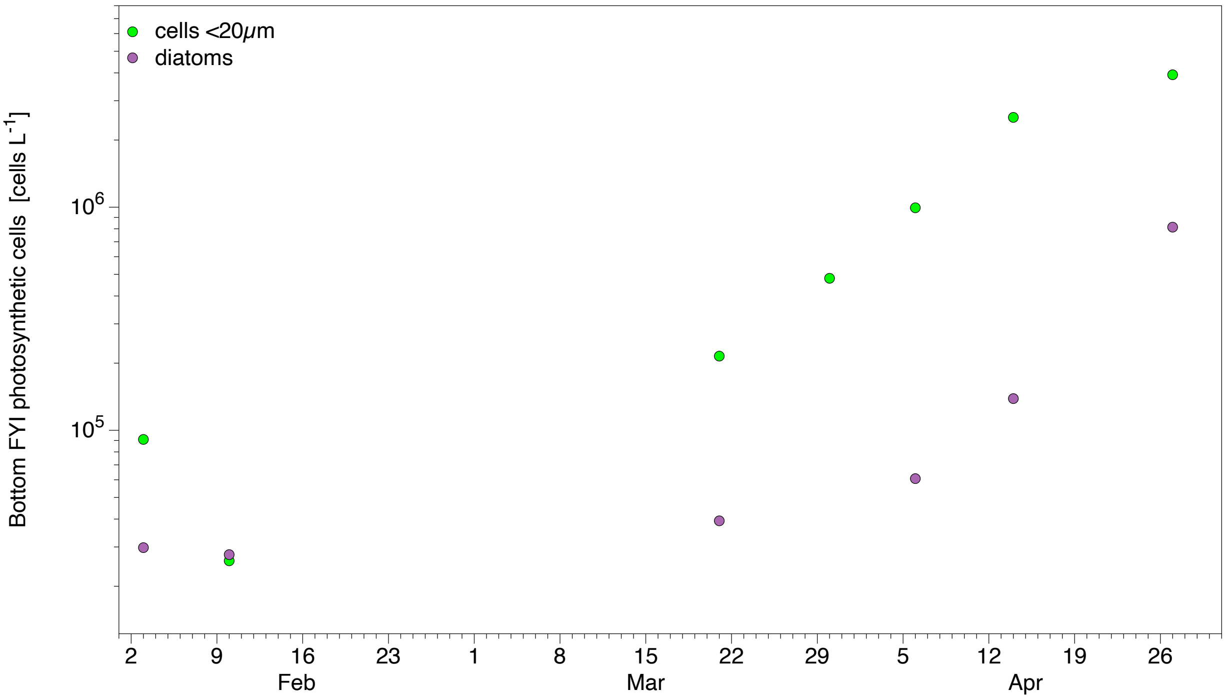

Figure S9: Temporal evolution of bottom ice photosynthetic cell counts.

Counts of bottom ice photosynthetic protist cells in the size range from 1 to 20 μm from bottom 5 cm sections of first year sea ice (FYI) based on flow cytometry (green symbols) and light-microscopic diatom counts (purple symbols) as a function of time.

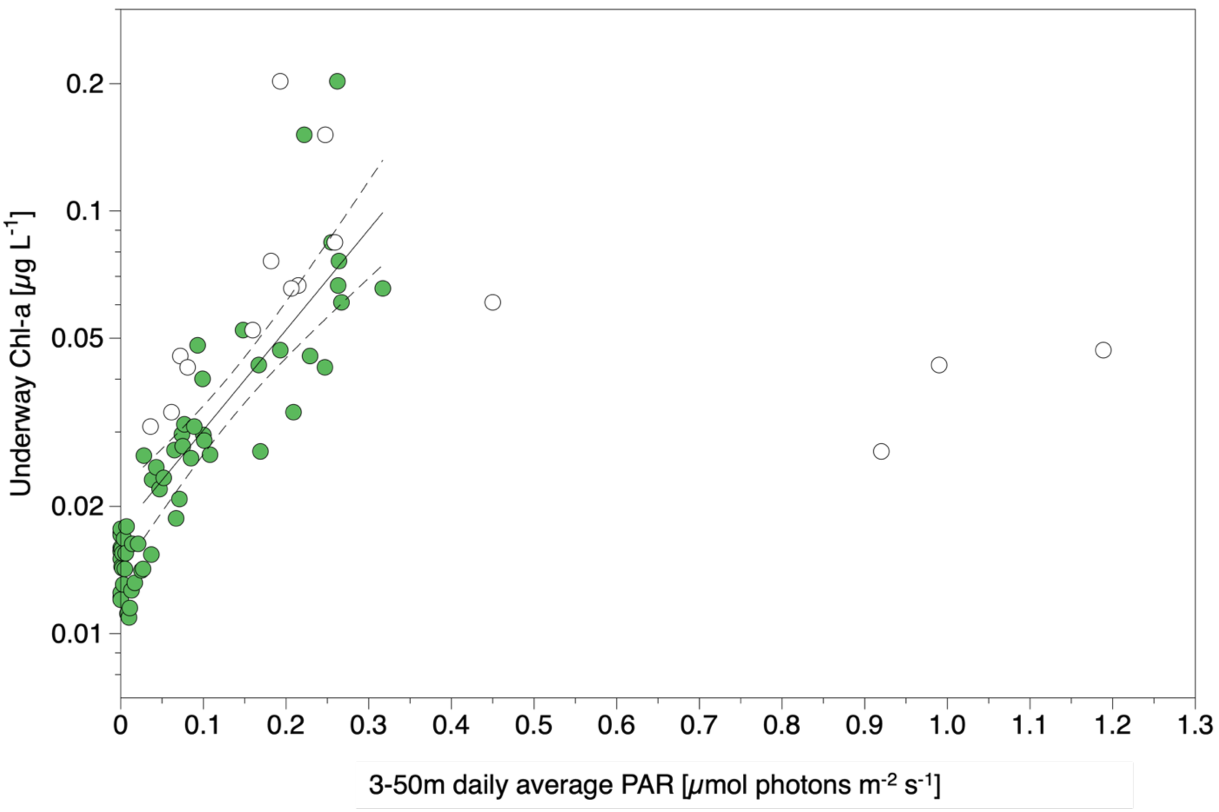

Figure S10: Relationship between Chl-a and light availability including the lead opening event.

Underway Chl-a concentrations (11 m depth; log scale) as a function of 3-50 m depth-integrated daily average PAR values from three light sensor strings (green symbols) with exponential fit ($r^2 = 0.66$; dashed lines indicate SE), excluding sensor gg after lead opening on April 17th (the latter displayed in open symbols).

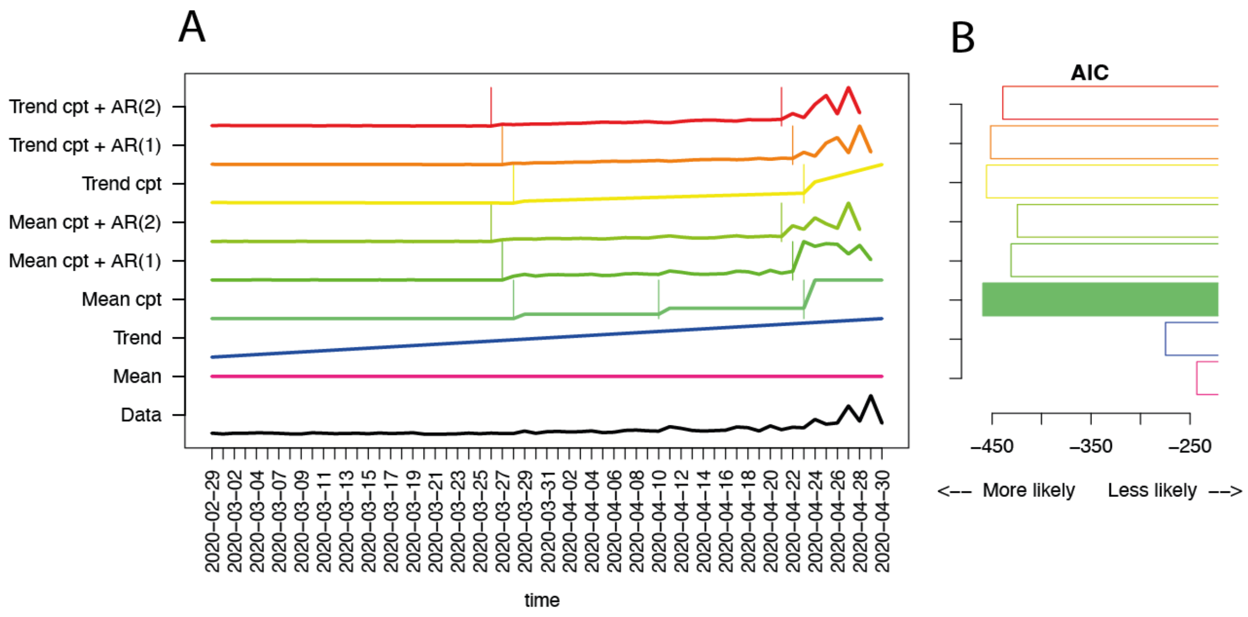

Figure S11: Change-point analysis for underway Chl-a data.

Results from change-point analysis displaying (A) the time series describing the data and the different statistical models, with vertical lines indicating the detected change-points; (B) the Akaike Information Criterion, which allows us to estimate the best performing model (the lowest value corresponds to the best performance).

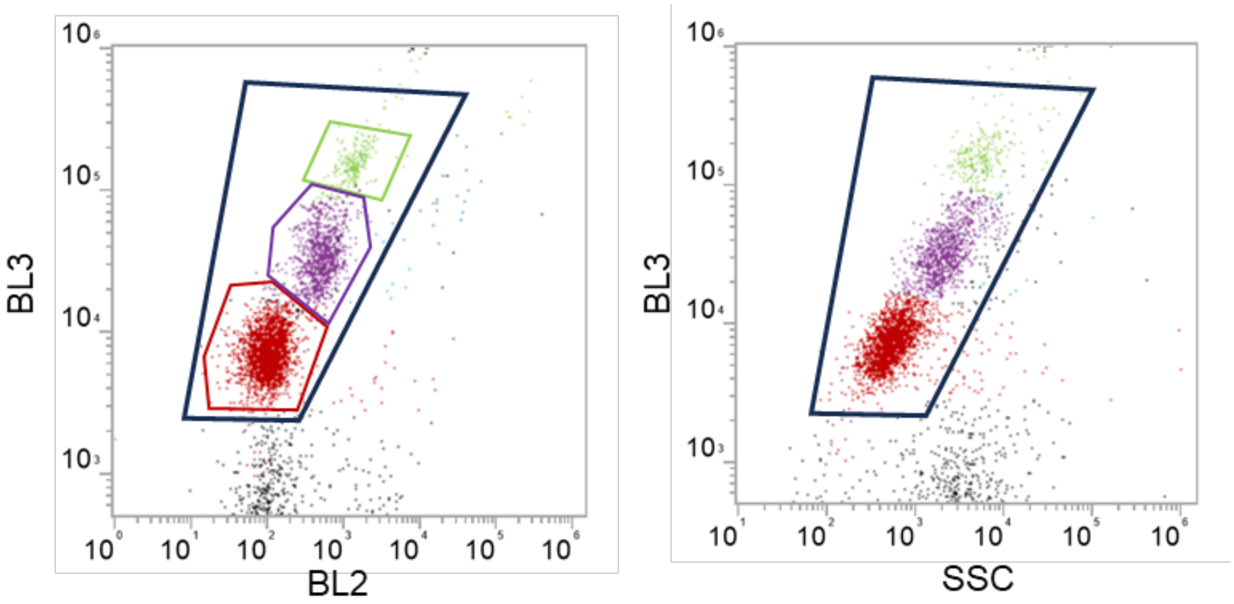

Figure S12: Gating strategy for flow cytometry samples.

Biparametric flow cytometry plots displaying the applied gating of the different phytoplankton groups, with the black frame highlighting all detected photosynthetic organisms in the size range of 1 and 20 µm. Different groups are indicated by the colored frames (red: picophytoplankton [1-2 µm]; purple: small nanophytoplankton [2-5 µm] and green: large nanophytoplankton [5-20 µm]) according to their grouping on either BL3 (red fluorescence) vs. BL2 (orange fluorescence) or BL3 (red fluorescence) vs. SSC (side scatter), similar to gating strategies as presented in Thyssen et al.[7].

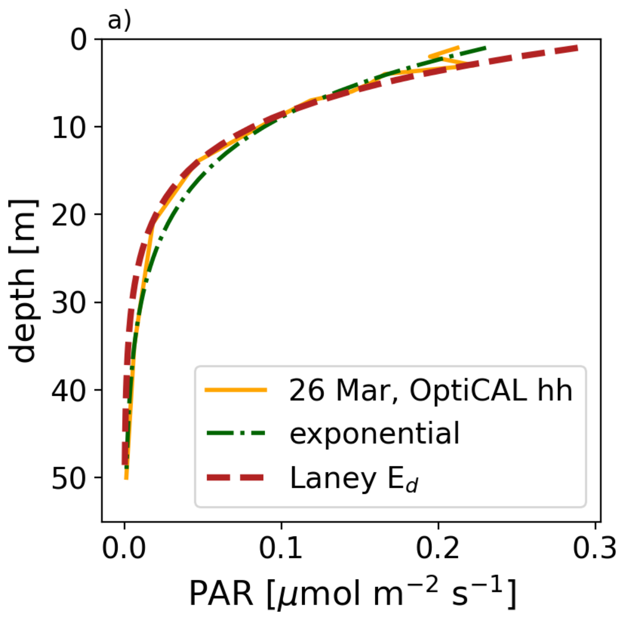

Figure S13: Example under-ice PAR profiles collected by OptiCAL.

On 26 March 2020 (a), the observed PAR profile (yellow, solid line) could be corrected for near-surface shading with the Laney model[8] (red, dashed line). Below 20 m depth, the model underestimates irradiance. On 10 April (b), the model fit was detected as invalid and therefore the observed profile was directly averaged. Both profiles indicate that a pure exponential fit was not able to provide a good estimate of the maximum available light under the ice.

Table S1: Taxonomic composition of protist assemblages.

Results from light-microscopy-based cell counts per liter of sample from 20 m water depth and from the 10 cm bottom sections of first year ice for the functional groups of centric and pennate diatoms, dinoflagellates, and other flagellates, as well as counts of important genera/families in the water column (*Pseudo-nitzschia* spp. and Gymnodiniaceae) and in the sea ice (*Nitzschia* spp. and *Navicula* spp.).

Environment	Date	all cells cells L^{-1}	diatoms cells L^{-1}	pennates cells L^{-1}	centrics cells L^{-1}	dinoflagellates cells L^{-1}	other flagellates cells L^{-1}	*Pseudo-nitzschia* spp cells L^{-1}	*Nitzschia* spp. cells L^{-1}	*Navicula* spp. cells L^{-1}	Gymnodiniaceae cells L^{-1}
Water	2020-03-06	15280	300	260	40	3060	2940	120	0	0	2940
Water	2020-03-14	17400	180	160	20	3820	5000	160	0	0	3700
Water	2020-03-22	35720	480	440	40	4400	11560	380	0	0	4180
Water	2020-03-27	13520	740	620	120	4440	4480	580	0	0	4280
Water	2020-04-05	19360	260	240	20	6580	6260	160	20	0	6460
Water	2020-04-17	38940	360	360	0	9500	8960	320	0	0	9120
Water	2020-04-23	29700	380	340	40	6320	7920	320	0	0	6120
Water	2020-04-30	20020	1880	1860	20	6720	2680	1760	0	0	6400
Water	2020-05-16	26960	4180	4160	20	2500	8580	3920	0	0	2260
Ice	2020-02-10	27717	5793	5455	338	4052	17872	602	1608	838	3883
Ice	2020-02-03	29788	10220	10220	0	1901	17425	2546	2328	1142	1402
Ice	2020-03-21	39286	7181	7131	50	1623	30062	1371	2662	940	1427
Ice	2020-04-06	60731	23823	23039	783	9227	26871	7121	6996	3106	8535
Ice	2020-04-14	138697	48504	46823	1680	11237	77180	7714	9179	9478	10421
Ice	2020-04-27	813696	439314	429544	9769	57483	313844	261760	60866	25391	51304

References

1. Beaulieu, C. & Killick, R. Distinguishing Trends and Shifts from Memory in Climate Data. *J. Clim.* **31**, 9519–9543 (2018).
2. Killick, R., Beaulieu, C., Taylor, S. & Hullait, H. Envcpt: Detection of structural changes in climate and environment time series. Version 1.1.3, available under https://github.com/rkillick/EnvCpt/
3. Shupe, M. D. *et al.* Overview of the MOSAiC expedition: Atmosphere. *Elem. Sci. Anthr.* **10**, 00060 (2022).
4. Fuchs, N. A multidimensional analysis of sea ice melt pond properties from aerial images. (2023) doi:10.26092/elib/2249.
5. Rabe, B. *et al.* Overview of the MOSAiC expedition: Physical oceanography. *Elem. Sci. Anthr.* **10**, 00062 (2022).
6. Boss, E. & Behrenfeld, M. In situ evaluation of the initiation of the North Atlantic phytoplankton bloom. *Geophys. Res. Lett.* **37**, (2010).
7. Thyssen, M. *et al.* Interoperable vocabulary for marine microbial flow cytometry. *Front. Mar. Sci.* **9**, (2022).
8. Laney, S. R., Krishfield, R. A. & Toole, John. M. The euphotic zone under Arctic Ocean sea ice: Vertical extents and seasonal trends. *Limnol. Oceanogr.* **62**, 1910–1934 (2017).
