# Supplementary material for: Photosynthetic light requirement near the theoretical minimum detected in Arctic microalgae
Source: Nat Commun. 2024 Sep 4;15:7385. doi: 10.1038/s41467-024-51636-8 (PMC11375000; doi:10.1038/s41467-024-51636-8)
Supplement: Supplementary file 3 — Reporting Summary [file 41467_2024_51636_MOESM3_ESM.pdf]

nature portfolio

Corresponding author(s): C. J. M. Hoppe

Last updated by author(s): 7/17/2024

Reporting Summary

Nature Portfolio wishes to improve the reproducibility of the work that we publish. This form provides structure for consistency and transparency in reporting. For further information on Nature Portfolio policies, see our Editorial Policies and the Editorial Policy Checklist.

Statistics

For all statistical analyses, confirm that the following items are present in the figure legend, table legend, main text, or Methods section.

n/a	Confirmed	
☐	☒	The exact sample size (*n*) for each experimental group/condition, given as a discrete number and unit of measurement
☐	☒	A statement on whether measurements were taken from distinct samples or whether the same sample was measured repeatedly
☐	☒	The statistical test(s) used AND whether they are one- or two-sided<br>*Only common tests should be described solely by name; describe more complex techniques in the Methods section.*
☒	☐	A description of all covariates tested
☒	☐	A description of any assumptions or corrections, such as tests of normality and adjustment for multiple comparisons
☐	☒	A full description of the statistical parameters including central tendency (e.g. means) or other basic estimates (e.g. regression coefficient) AND variation (e.g. standard deviation) or associated estimates of uncertainty (e.g. confidence intervals)
☐	☒	For null hypothesis testing, the test statistic (e.g. *F*, *t*, *r*) with confidence intervals, effect sizes, degrees of freedom and *P* value noted<br>*Give P values as exact values whenever suitable.*
☒	☐	For Bayesian analysis, information on the choice of priors and Markov chain Monte Carlo settings
☒	☐	For hierarchical and complex designs, identification of the appropriate level for tests and full reporting of outcomes
☒	☐	Estimates of effect sizes (e.g. Cohen's *d*, Pearson's *r*), indicating how they were calculated

Our web collection on statistics for biologists contains articles on many of the points above.

Software and code

Policy information about availability of computer code

Data collection	na
Data analysis	In order to statistically estimate the change point at which chlorophyll a started to accumulate, we used the R package “Detection of Structural Changes in Climate and Environment Time Series" ("EnvCPT", Beaulieu and Killick, 2018; Beaulieu et al. 2021), version 1.1.3. The Python code to process PAR data in the descripted way is available under https://zenodo.org/doi/10.5281/zenodo.12772363.

For manuscripts utilizing custom algorithms or software that are central to the research but not yet described in published literature, software must be made available to editors and reviewers. We strongly encourage code deposition in a community repository (e.g. GitHub). See the Nature Portfolio guidelines for submitting code & software for further information.

Data

Policy information about availability of data

All manuscripts must include a data availability statement. This statement should provide the following information, where applicable:

- Accession codes, unique identifiers, or web links for publicly available datasets
- A description of any restrictions on data availability
- For clinical datasets or third party data, please ensure that the statement adheres to our policy

The data generated in this study have been or will be deposited in the PANGAEA repository (https://www.pangaea.de/). Cruise track data used in Figure S1 is available as Kanzow (2020 65, doi: 10.1594/PANGAEA.924681). Continuous light data from OptiCALs are available as Anderson et al., (2023a66, doi: 10.1594/

PANGAEA.928495), Anderson et al., (2023b57, doi: 10.1594/PANGAEA.955045), and Anderson et al., (2023c67, doi: 10.1594/PANGAEA.954849). Continuous light data from the Light Harp are available as Fuchs et al., (2023a,b56,63, doi: 10.1594/PANGAEA.951614).
Chl-a concentration data from the underway system are available as Hoppe et al., (2023a68, doi: 10.1594/PANGAEA.963277) and the CTD rosette casts are available as Hoppe et al., (2023b69, doi: 10.1594/PANGAEA.962597). Flow cytometric cell count data from the water column are are available as Müller et al., (2023a70, doi: 10.1594/PANGAEA.963430), and data from sea ice are availble as Müller et al., (2023b71, doi: 10.1594/PANGAEA.963560). Data on microscopic cell counts are avaible as Kraberg (202472, doi: 10.1594/PANGAEA.965913); the summarized data as used in this study are provided in the Supplementary Information (Table S1). Data on Net Primary Production and Particulate Organic Carbon to Chl-a ratios will be submitted to PANGAEA. Source data for these and all other shown data are provided with this paper.

Research involving human participants, their data, or biological material

Policy information about studies with human participants or human data. See also policy information about sex, gender (identity/presentation), and sexual orientation and race, ethnicity and racism.

Reporting on sex and gender	This research does not involve human participants, their data, or biological material
Reporting on race, ethnicity, or other socially relevant groupings	This research does not involve human participants, their data, or biological material
Population characteristics	This research does not involve human participants, their data, or biological material
Recruitment	This research does not involve human participants, their data, or biological material
Ethics oversight	This research does not involve human participants, their data, or biological material

Note that full information on the approval of the study protocol must also be provided in the manuscript.

Field-specific reporting

Please select the one below that is the best fit for your research. If you are not sure, read the appropriate sections before making your selection.

☐ Life sciences ☐ Behavioural & social sciences ☒ Ecological, evolutionary & environmental sciences

For a reference copy of the document with all sections, see nature.com/documents/nr-reporting-summary-flat.pdf

Ecological, evolutionary & environmental sciences study design

All studies must disclose on these points even when the disclosure is negative.

Study description	The study consists of different field data collected during the MOSAiC campaign. The aim of the sudy was to combine biological and physical data to investigate the timing and lowest light use effiency of Arctic primary producers at the onset of the Arxtic growing season during the winter to spring transition period.
Research sample	Water samples were collected either from RV Polarsterns underway system (underway Chl-a), sea ice cores (sea ice Chla, NPP, cell counts) or the CTD rosette system (all other biological parameters). Irradiance measurements were conducted with autonomous sensors frozen into the seaice. We did not specifically study a certain organism group, but the natural protist community present at the time of sampling
Sampling strategy	We aimed at a daily sampling strategy for the underway samples, and a weekly sampling strategy for all other discrete samples. However, due to logistical challenges (weather, ice dynamics, team handover), not all sampling sites were accessible on a weekly basis, which is particularly the case for sea ice sampling. Sample size was determined based on logistical constrains.
Data collection	Samples from the upper mixed layer were collected approximately once per week at 20 m depth from Niskin bottles either from the ship's CTD rosette, or a smaller rosette deployed on the ice floe. Additional upper mixed layer samples with higher temporal resolution were taken daily from the ship's underway system with an intake at 11 m water depth. Sea ice samples were collected from level first-year ice (FYI) with a Kovacs Mark II 9 cm corer from a site located approx. 1 km away from the ship, where light pollution from the ship was not measurable during the polar night. Light data were collected from two different autonomous instrument setups measuring vertically resolved downwelling irradiance as a proxy for downward planar irradiance. OptiCALs measured in the water column, while the lightharp measured in the ice.
Timing and spatial scale	Data and saples were collected over the winter to spring transition period, i.e. between 2020-01-20 and 2020-04-30. Temporal resolution varies between daily (average light levels), near-daily (underway Chl-a concentrations) to about weekly (CTD rosette) and weekly to monthly (sea ice coring). Samples were collected while drifting through the central Arctic, the drift locations over the course of the sampling can be found in Figure S1. Relative sampling locations on drifting ice floe can be found in Figure S2.
Data exclusions	Irradiance measurements from one of the four light sensors during a lead opening event was excluded from further analysis. Analysis including all data is provided in the Supplement.
Reproducibility	no experimental data

Randomization	no experimental data
Blinding	not applicable

Did the study involve field work? ☐ Yes ☐ No

Field work, collection and transport

Field conditions	The samples were collected during the winter to spring transition. 2m air temperatures varied between -15 and -35°C (Shupe et al. 2022, Elementa) and surface ocean water temperatures were about -1.7°C (Rabe et al. 2022, Elementa). Sea ice thickness was about 1.5 to 2m (Lei et al. 20222, Elementa).
Location	The samples were collected in the ice-covered Eurasian basin of the central Arctic Ocean, between 88° and 84°N.
Access & import/export	The MOSAiC Campaign required 10 years of planning and a larger international consortium (20 nations) to allow for a one year drift campaign, thereby allowing to study the central Arctic Ocean in winter time when ice is usually too thick to allow ship-based expedition to reach the area. Given the central Arctic Ocean is not under regulation by local, national and international laws, no permits were required for this
Disturbance	Grey water was treated until considered void of traces of human activity, and re-salinated before release at 150m depth. Light contamination was limited to the bare minimum needed for safety. All recoverable installations on the ice were collected and returned to shore, but some frozen in installations remained in the ice (e.g., the LightHarp). Sample handing and processing was conducted on the ship.

Reporting for specific materials, systems and methods

We require information from authors about some types of materials, experimental systems and methods used in many studies. Here, indicate whether each material, system or method listed is relevant to your study. If you are not sure if a list item applies to your research, read the appropriate section before selecting a response.

Materials & experimental systems

n/a	Involved in the study	
☒	☐	Antibodies
☒	☐	Eukaryotic cell lines
☒	☐	Palaeontology and archaeology
☒	☐	Animals and other organisms
☒	☐	Clinical data
☒	☐	Dual use research of concern
☒	☐	Plants

Methods

n/a	Involved in the study	
☒	☐	ChIP-seq
☐	☒	Flow cytometry
☒	☐	MRI-based neuroimaging

Plants

Seed stocks	na
Novel plant genotypes	na
Authentication	na

Flow Cytometry

Plots

Confirm that:

- [x] The axis labels state the marker and fluorochrome used (e.g. CD4-FITC).
- [x] The axis scales are clearly visible. Include numbers along axes only for bottom left plot of group (a 'group' is an analysis of identical markers).
- [ ] All plots are contour plots with outliers or pseudocolor plots.
- [x] A numerical value for number of cells or percentage (with statistics) is provided.

Methodology

Sample preparation

Samples for flow cytometric analysis were taken in triplicates or quadruplicates of 1.8 mL of sample water and fixed with 36 µL 25 % glutaraldehyde (0.5 % final concentration) at 4 °C in the dark for approximately 2 hours, then flash frozen in liquid nitrogen and stored at -80 °C until analysis.

Instrument

Attune® NxT, Acoustic Focusing Cytometer (Invitrogen by Thermo Fisher Scientific) with a 20 mW 488 nm (blue) laser

Software

Attune® NxT software v3.1.2

Cell population abundance

Autotrophic pico-and nano-sized plankton were counted directly after thawing and the various groups discriminated based on their red fluorescence (BL3) vs. orange fluorescence (BL2), red fluorescence (BL3) vs. side scatter (SSC) and orange fluorescence (BL2) vs. side scatter (SSC). No cell sorting was performed.

Gating strategy

Autotrophic pico-and nano-sized plankton in the size range of 1 to 20 µm were discriminated based on their red fluorescence (BL3) vs. orange fluorescence (BL2), red fluorescence (BL3) vs. side scatter (SSC) and orange fluorescence (BL2) vs. side scatter (SSC). Names of size groups of photosynthetic and heterotrophic organisms are in accordance to "Standards and Best Practices For Reporting Flow Cytometry Observations: a technical manual (Version 1.1)" (Neeley et al., 2023). A short summary is listed here: RedPico = picophytoplankton (1-2 µm); RedNano = Nanophytoplankton (2-20µm), which includes subgroups RedNano_small (2-5 µm), RedNano_large (5-20 µm); OraPico = Nanophytoplankton with more orange fluorescence; OraNano = Cryptophytes; OraPicoProk = Synechococcus. Further, exemplary plots showing the gating strategies that were followed can be found in "Interoperable vocabulary for marine microbial flow cytometry" (Thyssen et al., 2022).

Flow cytometric contour plots are not included in the manuscript, when used for analysis all above checked points were applied. A figure exemplifying the gating strategy will be included in the reviseed manuscript.

Neeley, Aimee; Soto, Inia; Proctor, Christopher W (2023): Standards and Best Practices For Reporting Flow Cytometry Observations: a technical manual. Version 1.1. UNESCO/IOC, https://doi.org/10.25607/OBP-1864.2

Thyssen, Melilotus et al.,: Interoperable vocabulary for marine microbial flow cytometry. Front. Mar. Sci., https://doi.org/10.3389/fmars.2022.975877.

- [x] Tick this box to confirm that a figure exemplifying the gating strategy is provided in the Supplementary Information.
